# The effect of C_60_ fullerene on the *musculus gastrocnemius* contraction in chronically alcohol-exposed rats and the potential mechanism of its action

**DOI:** 10.1038/s41598-025-16765-0

**Published:** 2025-09-23

**Authors:** Olexandr Motuziuk, Dmytro Nozdrenko, Svitlana Prylutska, Nina Tverdokhleb, Mykola Petrovsky, Sergiy Vyzhva, Yuriy Prylutskyy, Peter Scharff, Uwe Ritter

**Affiliations:** 1 Faculty of Biology and Forestry, Lesya Ukrainka Volyn National University, Lutsk, 43025 Ukraine; 2 ESC “Institute of Biology and Medicine”, Taras Shevchenko National University of Kyiv, Kyiv, 01601 Ukraine; 3 Faculty of Plant Protection, Biotechnology and Ecology, National University of Life and Environmental Science of Ukraine, Kyiv, 03041 Ukraine; 4 Institute for Materials Science and Max Bergmann Center for Biomaterials, TUD Dresden University of Technology, 01062 Dresden, Germany; 5 Institute of Chemistry and Biotechnology, Technical University of Ilmenau, 98693 Ilmenau, Germany

**Keywords:** *Musculus gastrocnemius*, Chronic alcohol intoxication, С_60_ fullerene, Biomechanical parameters of muscle contraction, Indicators of pro- and antioxidant balance in the blood, Computer simulation, Computational biology and bioinformatics, Drug discovery, Medical research, Neurology

## Abstract

Over 90% of consumed alcohol is metabolized through oxidative and non-oxidative pathways, producing highly reactive compounds capable of generating reactive oxygen species (ROS). ROS induce increased oxidative stress and lipid peroxidation, thereby disrupting the structural integrity of myocytes and the functions of skeletal muscles in general. It is hypothesized that biocompatible and bioavailable C_60_ fullerenes, as potent antioxidants, can effectively absorb ROS, normalizing the functional state of the muscular system during the chronic alcoholic myopathy (CAM). Here, for the first time, the effect of C_60_ fullerenes (oral daily dose of 1 mg/kg) administered together with alcohol (40% ethanol in drinking water) on the contractile activity of the *musculus gastrocnemius* in male Wistar rats (age 1 month, weight 170 ± 10 g; *n* = 36) during the development of CAM over 3, 6, and 9 months was analyzed using tensometry. Biochemical analysis was used to evaluate pro- and antioxidant balance indicators in the blood of alcohol-exposed animals under the influence of C_60_ fullerenes. Finally, the potential mechanism of action of C_60_ fullerenes under chronic alcohol intoxication in rats was analyzed using a computer simulation technique. The data obtained indicate an improvement in the studied biomechanical markers of contraction in alcohol-exposed *musculus gastrocnemius* with a range of 16–50 ± 3%. Additionally, significant biochemical changes were observed in the pro- and antioxidant balance in the blood of experimental rats, showing an improvement of 22–39 ± 2% due to the action of C_60_ fullerenes, compared to the alcohol-exposed group. It is demonstrated that C_60_ fullerene nanoparticles can bind ethanol molecules, thereby reducing the negative effects of alcohol on the functioning of the muscular system.

## Introduction

Chronic alcohol use disorder (AUD)^1^ is a disease characterized by a syndrome of addictive alcohol dependence, which leads to specific somatic, myopathic, and neurological disorders in the body. According to the WHO, more than 3.3 million people worldwide die annually due to AUD^2,3^. Notably, approximately 25% of all deaths associated with chronic AUD occur among young people aged 20–39 years^4^.

Alcoholic myopathy is recognized as a distinct pathological condition that arises from other manifestations of alcoholic disease, such as liver damage, myocardial injury, or alcoholic polyneuropathy^5^. Several clinical forms of alcoholic myopathy are identified as those developing against acute or chronic alcohol intoxication and are collectively referred to as “alcohol-induced muscle damage“^6^. The most prevalent form is chronic alcoholic myopathy (CAM), which occurs in 40–60% of individuals who abuse alcohol^7,8^. Alcohol reduces the differentiation potential of muscle stem cells and disrupts the regulation of the extracellular matrix, thereby impairing the muscle’s regenerative capacity^9^. CAM encompasses a complex array of biochemical, physiological, and structural changes in skeletal muscle resulting from prolonged alcohol intoxication, leading to loss of muscle strength and mass^10^. Many symptoms, including acute muscle weakness, tonic spasms, high levels of tremor, and myoglobinuria with a high risk of acute renal failure^11^ characterize CAM. Importantly, CAM is not accompanied by a significant increase in the level of the enzyme creatine phosphokinase in the blood, and electromyography does not reveal changes typical of primary muscle damage, such as reduced amplitude and duration of motor unit action potentials^12^which often complicates its clinical diagnosis. It has been shown that a morphological indication of CAM development is muscle fiber atrophy, with its severity proportional to the duration of alcohol consumption and weakly dependent on dosage^13,14^. Progressive muscle fiber atrophy can lead to significant loss of muscle mass^15^.

The development of CAM is partially attributed to altered redox states and an imbalance of endogenous antioxidants in the body^16^. These impairments lead to a reduction in ATP content in skeletal muscle and a diminished redox ratio (NAD⁺/NADH), accompanied by increased electron leakage, primarily from complex III (ubiquinone), which promotes the generation of mitochondrial reactive oxygen species (ROS) and reverse electron transport. Collectively, these processes contribute to mitochondrial dysfunction in skeletal muscle, suppressed protein synthesis, and enhanced autophagy^17^. Alcohol enhances the activity of cytochrome P450, which also facilitates the formation of ROS. Finally, ethanol metabolism in the liver produces highly reactive compounds, and their further metabolism is also linked to the generation of ROS^18^.

The effective inactivation of ROS in both in vitro and in vivo systems by biocompatible and bioavailable C_60_ fullerene nanoparticles presents promising opportunities for their extensive biomedical applications^19,20^. It was shown that C_60_ fullerenes have more potent antioxidant properties compared to natural antioxidants vitamins C and E^21^as well as the exogenous antioxidants N-acetylcysteine (NAC) and β-alanine^22^which are often used in sports medicine. Notably, mitochondria are the primary organelles responsible for the production of ROS in damaged skeletal muscles. Because of this, C_60_ fullerenes with mitochondrial targeting^23^ are powerful scavengers of ROS induced by muscle pathologies of various kinds^24,25^.

Previously, on the models of alcohol-exposed rats^26^ it was shown that simultaneous administration of alcohol (40% ethanol in drinking water) and C_60_ fullerenes (the optimal daily dose of 1 mg/kg) results in a higher level of *musculus gastrocnemius* force response correction compared to other methods of this drug administration (1 h before and 1 h after alcohol intake). In particular, this therapeutic approach reduces fluctuations in force response during maximum force effort (the so-called “tremor”). Moreover, C_60_ fullerenes, which were administered to rats together with alcohol, reduced the time occurrence of fatigue processes in *musculus gastrocnemius* during the long-term development of alcoholic myopathy and inhibited oxidative processes in muscle, thereby preventing its degradation^27^.

Therefore, this study aimed to analyze in detail the effect of C_60_ fullerenes administered along with alcohol for 3, 6, and 9 months on the contractile activity of the *musculus gastrocnemius* in rats by evaluating (1) the most informative biomechanical markers of muscle dysfunction and biochemical indicators of pro- and antioxidant balance in the blood, as well as (2) the potential mechanism of C_60_ fullerene nanoparticles action using a computer simulation technique.

## Results

### Weight of experimental animals

In the control group, the average body weight of the rats increased from 170 ± 10 g at the beginning of the experiment to 210 ± 11 g, 240 ± 13 g, and 290 ± 15 g at the end of months 3, 6, and 9, respectively. In the alcohol-exposed group, body weight increased from 170 ± 10 g to 200 ± 10 g, 220 ± 12 g, and 250 ± 13 g over the same time points. Finally, in the alcohol + C_60_-treated group, average body weight did not differ reliably from that of the alcohol-only group.

### Biomechanical analysis of *musculus gastrocnemius* contraction in alcohol-exposed rats

**Fig. 1:**
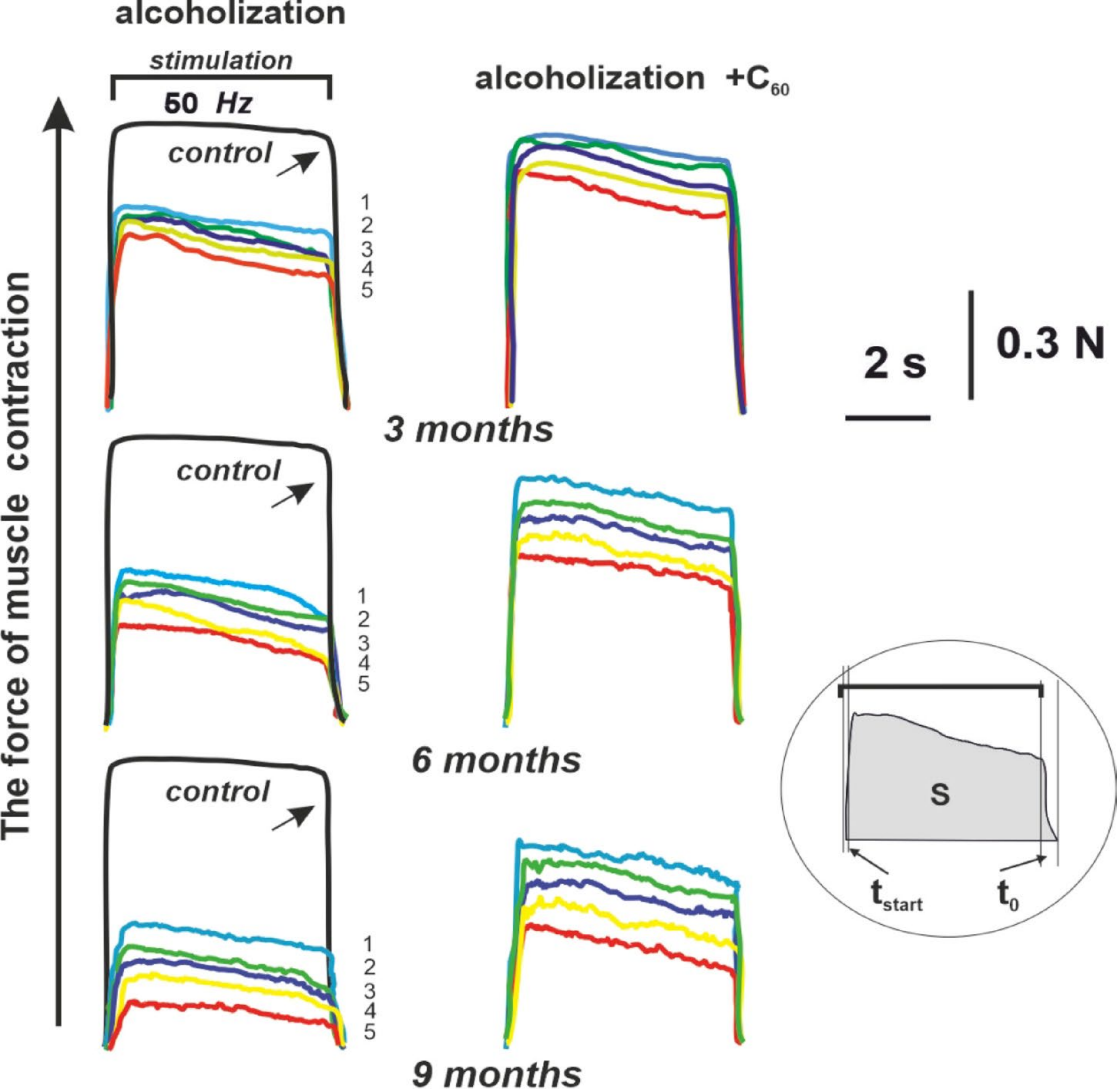
The strength (N, Newton) of 5 (1, 2, 3, 4, 5) consecutive contractions of the *musculus gastrocnemius* of experimental rats induced by non-relaxation stimulation pools of 50 Hz for 5 s: control – rats of the control group; alcoholization - alcohol-exposed rats; alcoholization + C_60_ – administration of C_60_ fullerene mixed with alcohol; 3, 6, 9 months – duration of the experiment 3, 6 and 9 months, respectively; S – integrated muscle power (an area under the force curve); t_start_ – time of the beginning of the muscle force response; t_0_ – time for the muscle force response to reach its initial level.

Muscle weakness is the most prevalent clinical manifestation of CAM^28^characterized by a reduction in muscle contractile force and altered timing in achieving force parameters that correspond to the frequency of efferent stimulation observed in an intact muscle. To assess these myopathic impairments in the *musculus gastrocnemius*, we applied a stimulation signal at a frequency of 50 Hz for a duration of 5 s (Fig. 1).

**Fig. 2:**
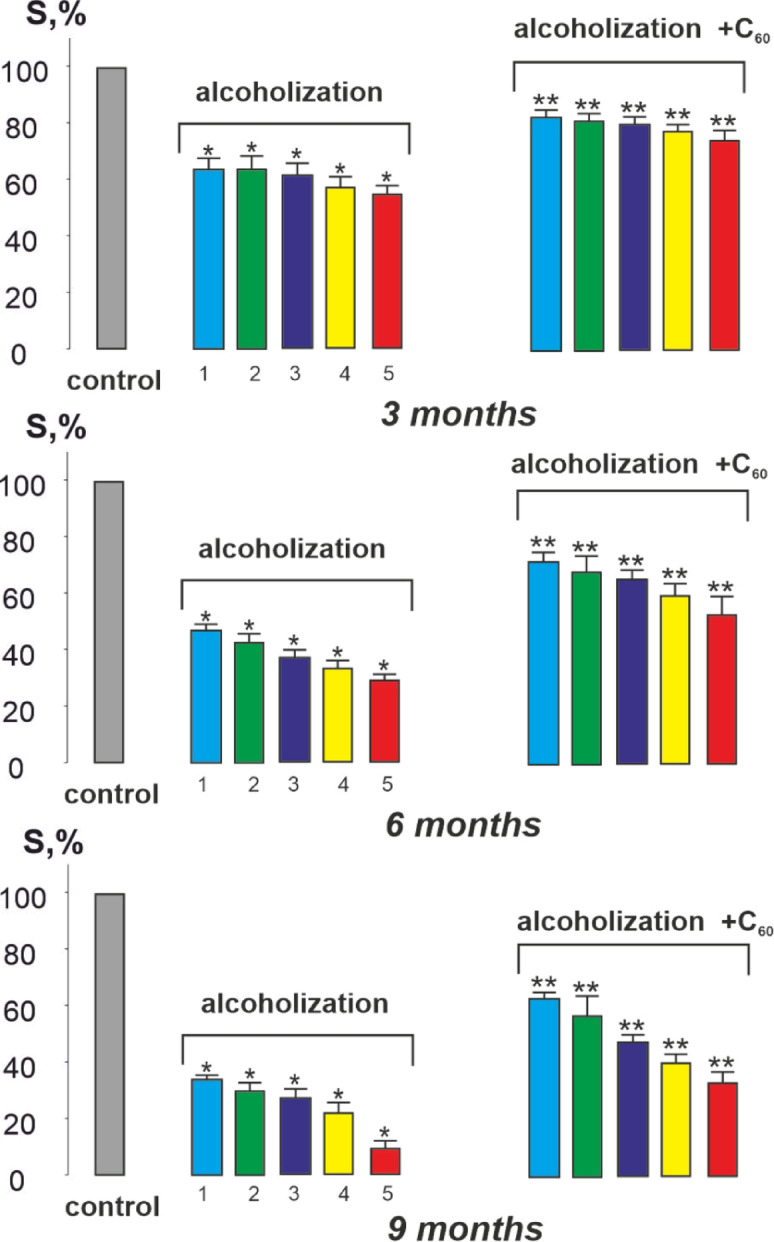
Integrated power of the *musculus gastrocnemius* (S): control – rats in the control group (S taken as 100%); alcoholization – alcohol-exposed rats; alcoholization + C_60_ – administration of C_60_ fullerene mixed with alcohol; 3, 6, 9 months - duration of experiment 3, 6 and 9 months, respectively; 1, 2, 3, 4, 5 - numbering of 5 consecutive contractions of the *muscle gastrocnemius*. *p < 0.05 vs. control group ((*n* = 36)); **p < 0.05 vs. alcohol-exposed group (*n* = 36; *n* = 12 animals in each subgroup: 3, 6, and 9 months).

Figure 2 shows that after 3 months of alcohol-exposed animals, the value of integrated muscle power, as a physiological analog of its performance^29^significantly decreased during all stimulation pools and at the 5th contraction amounted to 57 ± 3% of the control value. The use of C_60_ fullerene revealed an improvement of this parameter by 16 ± 1% compared to the alcohol-exposed group.

The integrated muscle power of rats after 6 months of AUD decreased to 29 ± 1% of the control at the 5th contraction. The use of C_60_ fullerene improved this indicator by 21 ± 1% compared to the alcohol-exposed group (Fig. 2).

Finally, AUD for 9 months significantly decreased the integrated muscle power, which amounted to 10 ± 1% of the control at the 5th contraction. The use of C_60_ fullerene increased this value by 23 ± 1% compared to the alcohol-exposed group (Fig. 2).

**Fig. 3:**
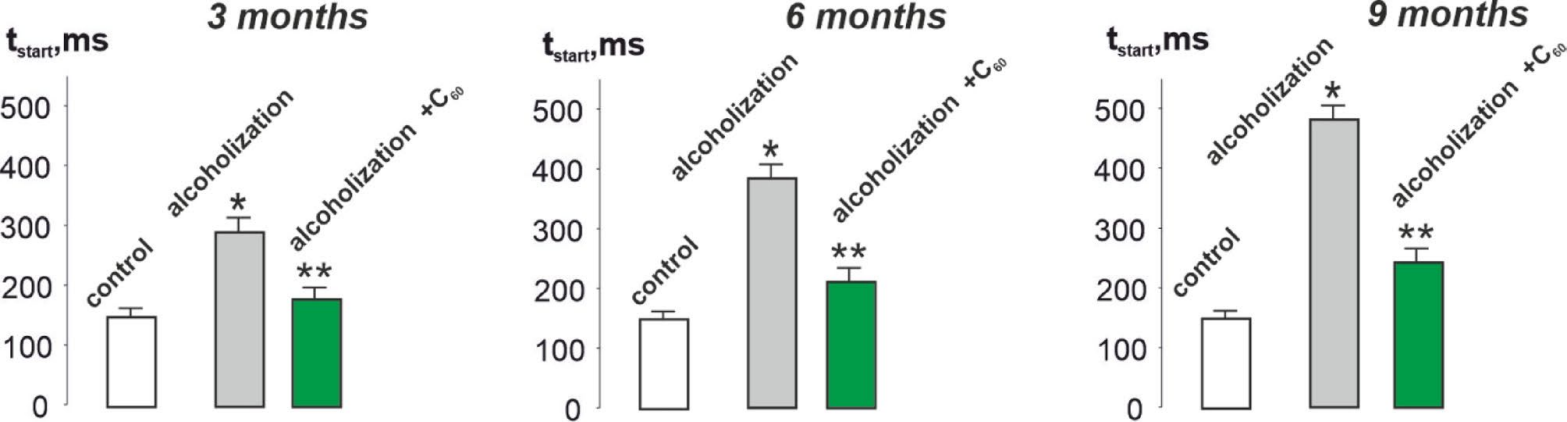
Time of the beginning of *musculus gastrocnemius* force response (t_start_): control – rats of the control group; alcoholization – alcohol-exposed rats; alcoholization + C_60_ – administration of C_60_ fullerene mixed with alcohol; 3, 6, 9 months – duration of the experiment 3, 6 and 9 months, respectively. *p < 0.05 vs. control group (*n* = 36); **p < 0.05 vs. alcohol-exposed group (*n* = 36; *n* = 12 animals in each subgroup: 3, 6 and 9 months).

As can be seen from Fig. 3, the time of the beginning of the muscle force response increased from 150 ± 15 ms in the control to 300 ± 20, 390 ± 15, and 480 ± 22 ms at 3, 6, and 9 months of alcohol consumption, respectively. The use of C_60_ fullerenes reduced this parameter to 185 ± 8, 210 ± 14, and 240 ± 16 ms, respectively, which was 38 ± 2%, 46 ± 3%, and 50 ± 4% compared to the alcohol-exposed group.

**Fig. 4:**
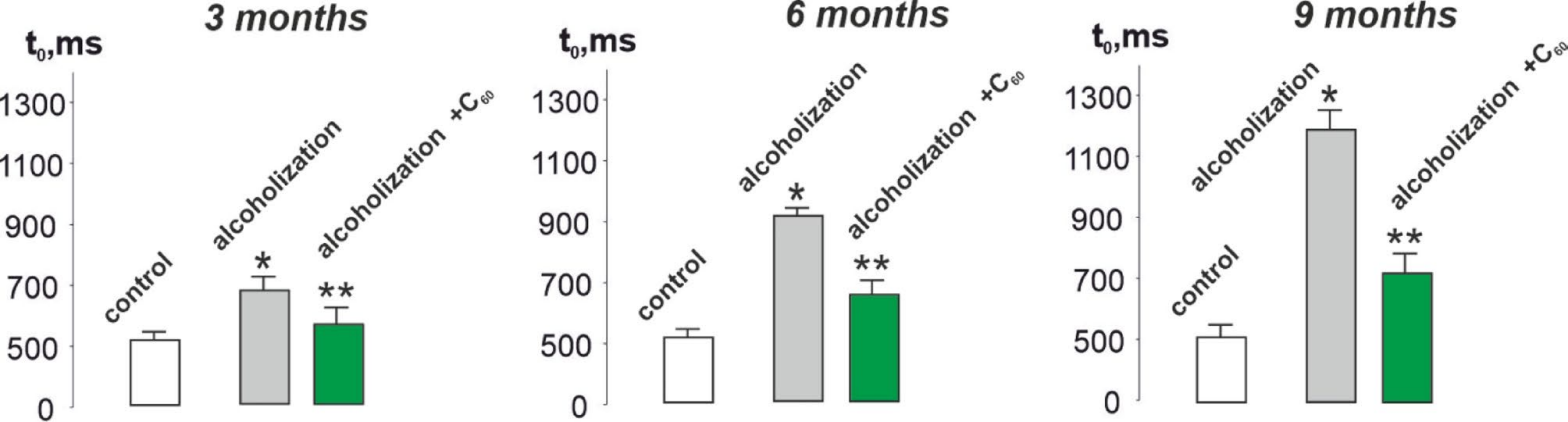
Time for the *musculus gastrocnemius* force response to reach its initial level (t_0_): control – rats of the control group; alcoholization – alcohol-exposed rats; alcoholization + C_60_ – administration of C_60_ fullerene mixed with alcohol; 3, 6, 9 months – duration of the experiment 3, 6 and 9 months, respectively. *p < 0.05 vs. control group (*n* = 36); **p < 0.05 vs. alcohol-exposed group (*n* = 36; *n* = 12 animals in each subgroup: 3, 6, and 9 months).

**Fig. 5:**
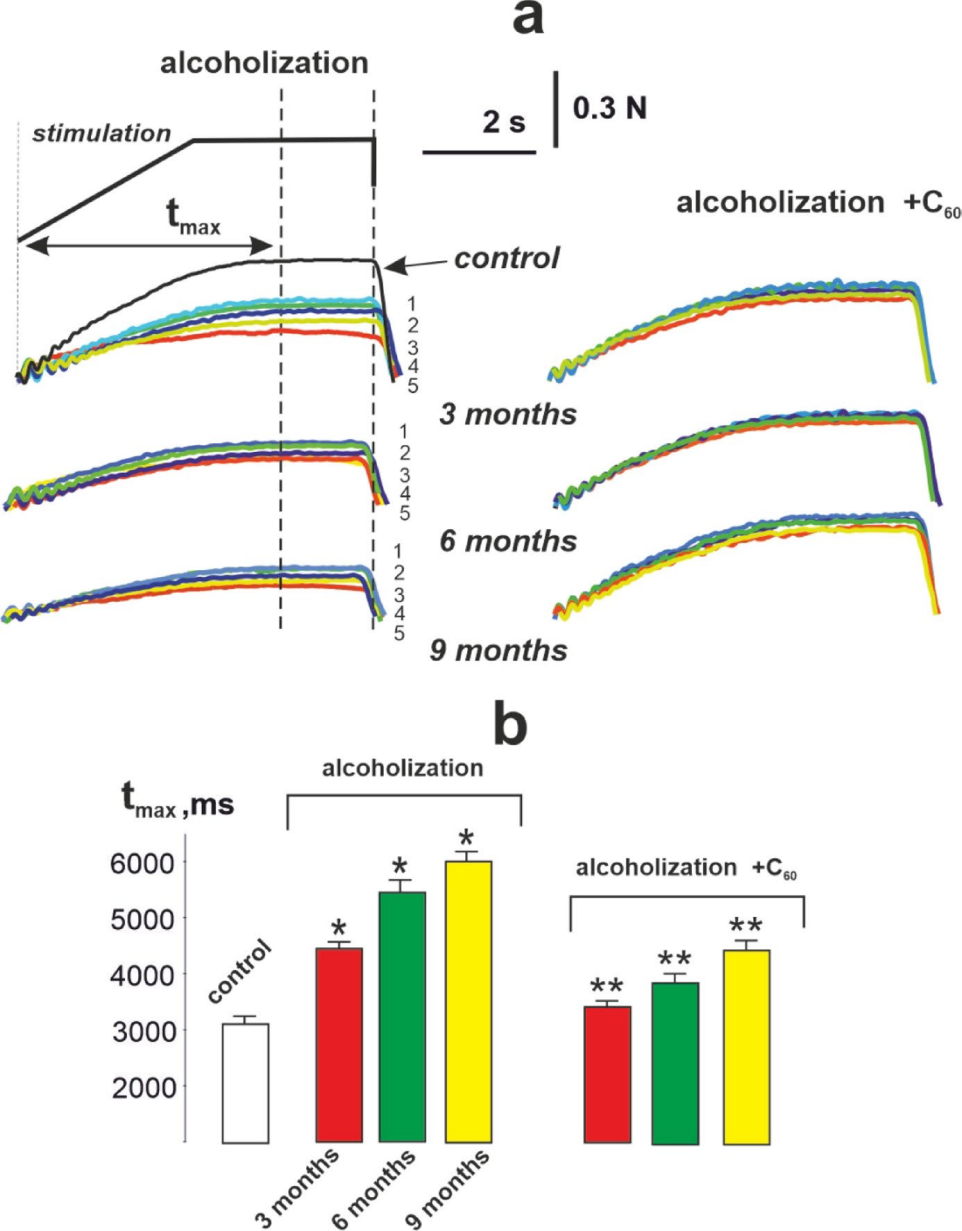
Mechanograms depicting the force response (N, Newton) of the *musculus gastrocnemius* of experimental rats elicited by 5 s of non-relaxation stimulation pools with an extended pre-tetanic contraction phase (**a**); t_max_ – time for the muscle contraction force to reach the maximum level (**b**): control – rats in the control group; alcoholization – alcohol-exposed rats; alcoholization + C_60_ – administration of C_60_ fullerene in conjunction with alcohol; 3, 6, 9 months – duration of experiment 3, 6 and 9 months, respectively; 1, 2, 3, 4, 5 - numbering of 5 consecutive contractions of the *musculus gastrocnemius*. *p < 0.05 vs. control group (*n* = 36); **p < 0.05 vs. alcohol-exposed group (*n* = 36; *n* = 12 animals in each subgroup: 3, 6, and 9 months).

As can be seen from Fig. 4, the time for the muscle force response to reach its initial level increased from 500 ± 17 ms in control to 690 ± 21, 900 ± 27 and 1200 ± 2 9 ms at 3.6 and 9 months of AUD, respectively. With the use of C_60_ fullerenes, this parameter decreased to 570 ± 9, 680 ± 11, and 710 ± 10 ms, respectively, which was 17 ± 1%, 24 ± 1%, and 41 ± 3% compared to the alcohol-exposed group.

To analyze the time for the muscle contraction force to reach the maximum level, we used a complex modulated stimulation signal with an extended time of the pre-tetanic phase of contraction lasting 3 s (Fig. 5a).

As illustrated in Fig. 5b, in the control group, the time for the muscle contraction force to reach its maximum level (3120 ± 28 ms) was consistent with the duration of pre-tetanic stimulation. After AUD for 3, 6, and 9 months, this parameter was 4530 ± 46, 5515 ± 52, and 6150 ± 55 ms, respectively. With the use of C_60_ fullerenes, it decreased to 3300 ± 29, 3820 ± 32 and 4200 ± 41 ms, respectively, which was 27 ± 1%, 31 ± 2%, and 32 ± 2% compared to the alcohol-exposed group.

**Fig. 6:**
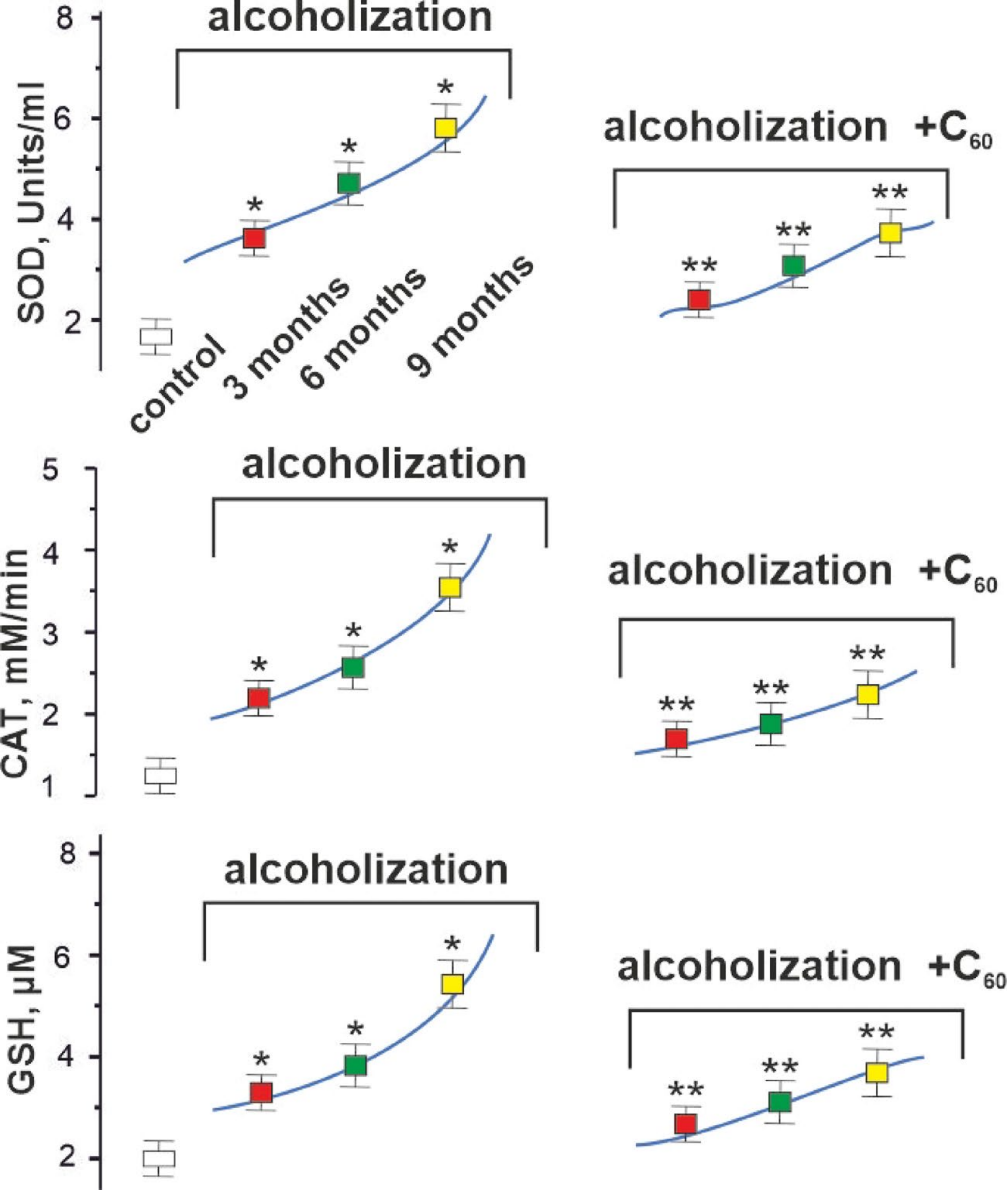
Parameters of pro- and antioxidant balance (SOD, CAT, and GSH) in the blood plasma of experimental rats: control – rats of the control group; alcoholization – alcohol-exposed rats; alcoholization + C_60_ – administration of C_60_ fullerene in a mixture with alcohol; 3, 6, 9 months – duration of the experiment 3, 6 and 9 months, respectively. *p < 0.05 vs. control group (*n* = 36); **p < 0.05 vs. alcohol-exposed group (*n* = 36; *n* = 12 animals in each subgroup: 3, 6, and 9 months).

### Biochemical analysis of blood in alcohol-exposed rats

To verify the antioxidant effect of C_60_ fullerenes through modification of ROS-dependent mechanisms and their effect on the activity of endogenous antioxidants, we analyzed the values of pro- and antioxidant balance (superoxide dismutase (SOD) and catalase (CAT) activities, reduced glutathione (GSH) concentration) in the blood plasma of alcohol-exposed rats (Fig. 6).

SOD is the most powerful natural antioxidant and the first-line enzyme of the body’s antioxidant defense. As can be seen from Fig. 6, the activity of SOD increased to 3.8 ± 0.3, 4.8 ± 0.4 and 5.9 ± 0.5 Units/ml in the case of 3, 6, and 9 months of AUD, respectively, and in the control it was 1.7 ± 0.1 Units/ml. The use of C_60_ fullerenes reduced these indicators to 2.4 ± 0.2, 3.0 ± 0.3 and 3.6 ± 0.3 Units/ml, respectively, which amounted to 37 ± 3%, 38 ± 3%, and 39 ± 3% compared to the alcohol-exposed group.

CAT works in conjunction with SOD by decomposing hydrogen peroxide into water and molecular oxygen, thereby preventing the formation of highly reactive hydroxyl radicals. The CAT activity increased to 2.2 ± 0.1, 2.5 ± 0.1 and 3.5 ± 0.2 mM/min in the case of 3, 6, and 9 months of AUD, respectively, and in the control group it was 1.3 ± 0.1 mM/min. The use of C_60_ fullerenes reduced these values to 1.7 ± 0.1, 1.9 ± 0.1 and 2.2 ± 0.1 mM/min, respectively, which amounted to 33 ± 3%, 34 ± 3%, and 37 ± 3% compared to the alcohol-exposed group (Fig. 6).

The cellular mechanisms of antioxidant defense are linked to the functioning of a powerful GSH link. Figure 6 shows that there is a significant increase in the concentration of GSH in the development of CAM, namely, it was 3.2 ± 0.2, 3.9 ± 0.2 and 5.5 ± 0.3 µM in the case of 3, 6, and 9 months of AUD, respectively, and in the control − 2.0 ± 0.1 µM. The use of C_60_ fullerenes reduced these indicators to 2.8 ± 0.1, 3.0 ± 0.2 and 3.8 ± 0.2 µM, respectively, which amounted to 22 ± 1%, 23 ± 1%, and 31 ± 3% compared to the alcohol-exposed group.

### Analysis of molecular dynamics (MD) simulation

Initially, MD simulations were performed on systems containing 1 to 4 С_60_ fullerenes in ethanol solutions. In these cases, complexes from 2 to 4 С_60_ molecules were destroyed with the appearance of single С_60_ fullerenes during simulation. Single С_60_ fullerenes don’t adsorb molecules of ethanol. The other data can be seen for the cases with 5, 10, 13, and 20 С_60_ fullerene units. These numbers of С_60_ fullerene created the stable, predominantly ellipsoidal nanoaggregates that don’t break down in MD running in the ethanol solution. Similar results are observed for С_60_ fullerenes in polar solvents^30–32^. 5 C_60_ fullerenes are placed at the tops of a triangular bipyramid, the maximal diagonal of which is 2.2 nm. Nanoaggregates consisting of 10 and 13 C_60_ fullerenes are almost spherical with diameters of 2.5 and 2.9 nm, respectively. The C_60_ fullerene cluster, constructed from 20 units, has an ellipsoidal shape with the longest principal axis of 3.4 nm and two others approximately equal to 2.5 nm. Distances represented here were measured between the farthest points of opposite C_60_ fullerene surfaces.

**Fig. 7:**
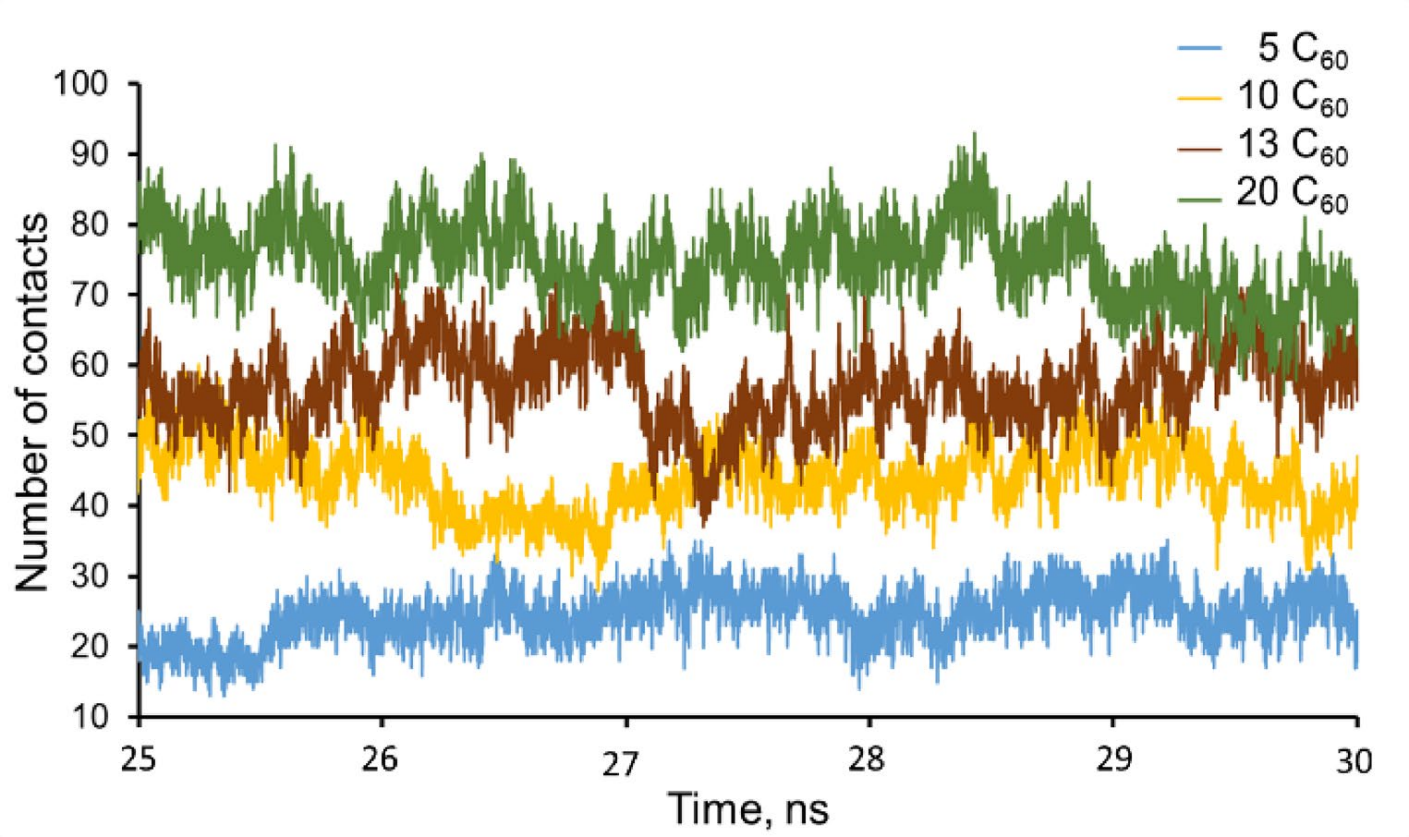
Contact numbers of the first solvation shell with С_60_ fullerene nanoaggregates during the last 5 ns of simulation.

**Fig. 8:**
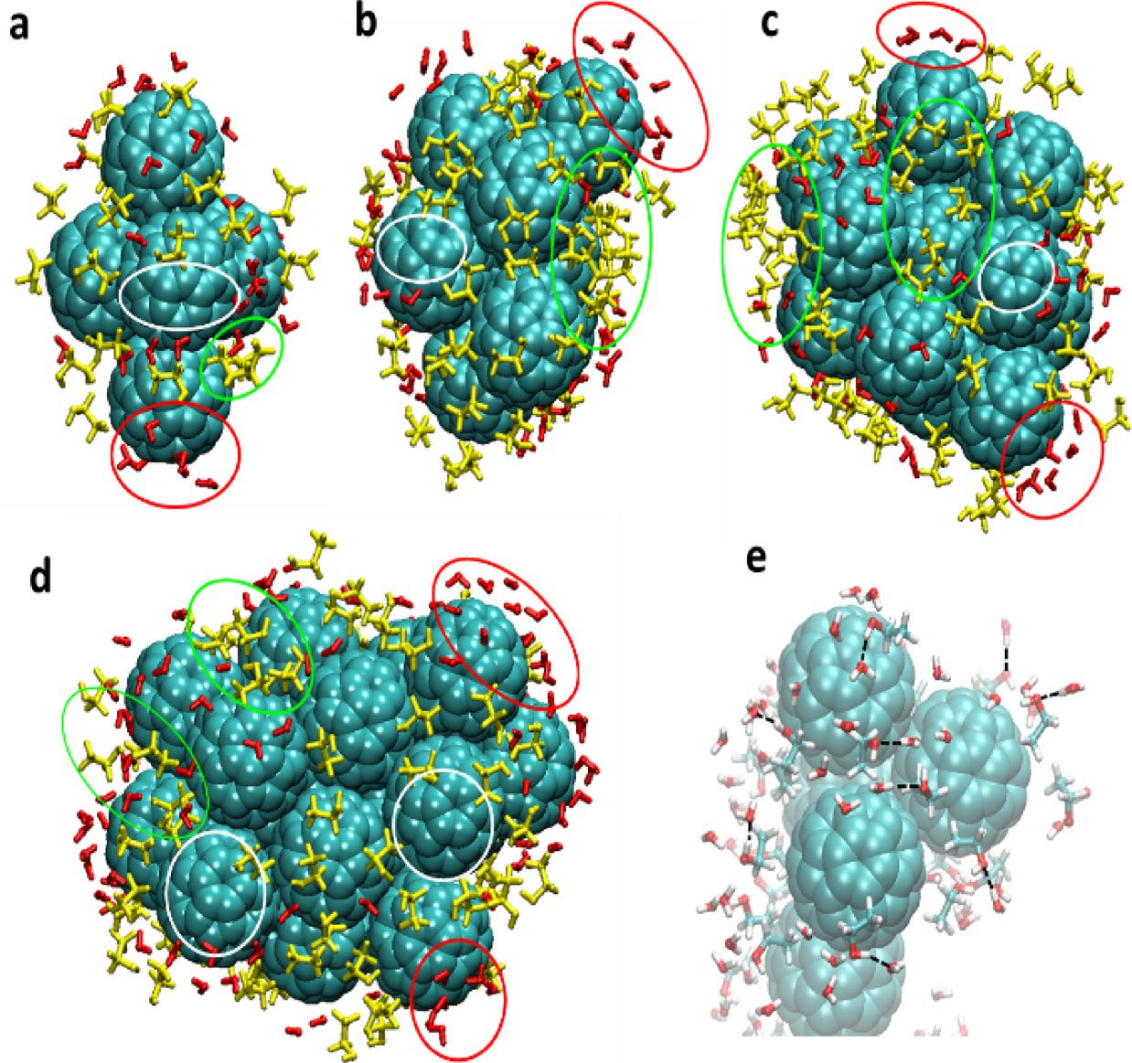
The first solvation shell of the 5, 10, 13, and 20 C_60_ fullerene nanoaggregates (**a**-**d**). Water molecules are red, ethanol is yellow. Circles show: white – “dry” regions, green – cavities between C_60_ fullerenes, and red – convex surfaces. Fragment of 5 C_60_ fullerene complex with first ethanol solvation shell, where the molecules are occupied by water within 0.24 nm (**e**). The black dotted lines demonstrate hydrogen bonds between ethanol OH-groups and water. Oxygen atoms are red, hydrogens – white, and carbons – cyan.

The minimal distance of the neighboring С_60_ fullerenes in clusters, directly interact with each other is 0.27 nm, and the maximum is 0.32 nm. The number of contacts between the С_60_ fullerene cluster and ethanol/water molecules at a distance of 0.32 nm (Fig. 7) was calculated. The averaged values of these contacts are represented in Table 1. On average, the number of contacts between clusters and water is only 23% larger than with ethanol, which means that during simulations, nanoaggregates adsorbed on their surface both ethanol and water molecules. Figure 8a–d shows the first solvation shell of С_60_ fullerene nanoaggregates consisting of 5, 10, 13, and 20 units. The shortest distance between the surface of С_60_ fullerene clusters and ethanol molecules is 0.24 nm, a few water molecules can touch some C_60_ fullerenes at a distance of 0.22 nm. Hence, ethanol molecules occupy clusters inhomogeneously: they tend to be located in the cavities between С_60_ fullerenes, whereas water mostly interacts with the convex surfaces of С_60_ fullerenes. Also, it is determined that some “dry” regions (Fig. 8), which is confirmed by conclusions in the study^33^.

Moreover, the majority of ethanol OH-groups orient to water molecules, tending to create hydrogen bonds with them (Fig. 8e). The net of these hydrogen bonds becomes thicker with the increase of the cluster size. In general, the amount of adsorbed ethanol molecules depends on the measure of С_60_ fullerene nanoaggregates (Fig. 7; Table 1).

**Table 1:** Average numbers of ethanol - С_60_ fullerene and water - С_60_ fullerene contacts (M ± SE).

Number of С_60_ fullerenes in nanoaggregates	Average number of ethanol - С_60_ fullerene contacts	Average number of water - С_60_ fullerene contacts
5	25 ± 4	29 ± 6
10	44 ± 5	67 ± 9
13	55 ± 6	74 ± 11
20	75 ± 6	93 ± 11

## Discussion

The results obtained from measuring the weight of the experimental animals showed that alcohol consumption reduced their weight gain to 15 ± 1% compared to the control group, which agrees well with previous data^10^. The use of water-soluble C_60_ fullerenes did not affect this process.

The biomechanical studies conducted revealed that the value of integrated muscle power (Fig. 2) decreases, and the values ​​of the time characteristics of *musculus gastrocnemius* contraction (Figs. 3, 4 and 5) increase, in the context of chronic alcohol intoxication in animals. These effects may be caused by impaired interaction of muscle myofilaments, an increase in their stiffness, dysfunction of the calcium pump, and the sarcoplasmic reticulum system^5,34,35^. One of the reasons for the above-described mechanokinetic effects may also be a decrease in the synthesis of myotic proteins. It has been shown^36^ that in the case of CAM, there is a decrease in the synthesis of not only the main contractile protein, myosin, but also cytoskeletal proteins (actin, troponin, titin, nebulin), which provide conditions for the interaction of actin and myosin. This not only changes the stiffness of myocytes but also extends the time for establishing a smooth tetanic muscle contraction. Furthermore, the use of water-soluble C_60_ fullerenes improved the studied biomechanical parameters by 16–50 ± 3% compared to the alcohol-exposed group. Thus, C_60_ fullerenes can mitigate damage to the main links of the “muscle excitation-response”, inhibiting the development of pathological processes in alcohol-dependent muscle cell damage.

The conducted biochemical studies showed (Fig. 6) that the blood plasma biomarkers associated with oxidative stress in experimental animals exhibited a significant increase against the background of CAM development. This indicates that the muscular system performs work that is too intense for its physiological level. The use of C_60_ fullerenes, possessing a strong antioxidant capability, improved the biochemical parameters by 22–39 ± 2% compared to the alcohol-exposed group. Therefore, the restoration of biochemical indicators achieved through C_60_ fullerene intervention demonstrates its capacity to disrupt the cycle of oxidative stress and mitochondrial dysfunction.

Finally, in addition to the antioxidant effect of C_60_ fullerenes, one of the explanations for their effective use in combination with alcohol may be the binding of ethanol molecules to C_60_ fullerene nanoparticles, excluding them from the metabolic cycle and thus reducing the development of CAM and improving the skeletal muscle contraction, which was confirmed by the model calculations (Fig. 8).

The mechanisms underlying the toxic effects of alcohol mediated by oxidative stress are complex and not yet fully elucidated. Ethanol has been shown to induce oxidative damage, including lipid peroxidation (LPO) of cellular membranes and mitochondrial dysfunction^37^. Ethanol metabolism involves several enzymatic pathways, including alcohol dehydrogenase, the microsomal ethanol oxidizing system (MEOS), and catalase. Each of these pathways generates free radicals that can compromise the body’s antioxidant defense system. Additionally, ethanol itself, hyperlactacidemia, and elevated NADH levels enhance xanthine oxidase activity, leading to increased superoxide production. Both LPO and superoxide generation are correlated with cytochrome P450 2E1 expression. MEOS contributes to oxidative stress both directly and indirectly by disrupting endogenous protective mechanisms^38^. These multifaceted systemic processes hinder the identification of the most influential factors in the development of CAM.

It has been demonstrated that incorporating the antioxidant NAC into the diet of alcohol-exposed rats enhances the activity of glutathione peroxidase and citrate synthase, thereby improving energy metabolism^39^. The addition of the antioxidant procysteine mitigated alcohol-induced oxidative stress compared to untreated rats^40^. In contrast, the addition of alpha-tocopherol did not prevent the decline in muscle protein synthesis in animals administered alcohol^34^. The use of well-known antioxidants such as carotenoids, vitamins C and E, selenium, polyphenols, and flavonoids to normalize oxidative stress in skeletal muscles affected by alcohol also yielded conflicting results^41^.

In summary, the following potential mechanisms of C_60_ fullerene action can be considered. Ethanol disperses from the bloodstream to all tissues and body fluids^42^. The ethanol concentration in a tissue is influenced by its relative water content. Similar to water, ethanol can permeate cell membranes^43^. Additionally, there are no proteins in blood plasma that bind to ethanol^44^. It is known that more than 90% of consumed alcohol is metabolized by oxidative and non-oxidative pathways, producing such reactive compounds as acetaldehyde, acetate, fatty acid ethyl ester, etc^18,45^. Within the framework of such processes, ROS is generated, which causes an increase in oxidative stress and LPO, thus disrupting the structural integrity of cells and the functions of tissues and organs. Increased ROS production reduces the level of endogenous antioxidants^46^. Therefore, it is assumed that, on the one hand, C_60_ fullerenes, as powerful exogenous antioxidants^19,21^can effectively absorb ROS and remove them from the body^47^normalizing the functional state of the muscular system against the background of alcoholic myopathy. On the other hand, stable-sized nanoparticles of C_60_ fullerenes present in ethanol can effectively bind its molecules and, thus, entering the body, reduce the negative effects of alcohol on the functioning of the muscular system.

## Мaterials and methods

### Preparation of water-soluble C_60_ fullerene

A water-soluble C_60_ fullerene with purity of more than 99.95% has been prepared according to the reproducible method^48,49^which is based on transferring C_60_ molecules from toluene solution into the aqueous phase by ultrasonic treatment. The used concentration of C_60_ fullerenes in water was 0.15 mg/ml. According to the data of high-resolution microscopy and light scattering spectroscopy^48^the resulting sample is a typical colloidal nanofluid, which mainly contains both individual C_60_ fullerenes and their nanoaggregates up to 100 nm. Moreover, it remains highly stable for up to 18 months at a temperature range of + 4–25 ^0^С.

### In vivo study

The rats come from the university’s breeding program (Institute of Biology and Medicine). All experiments were conducted on male Wistar rats aged 1 month with a body weight of 170 ± 10 g^26,27,50^. The rats were housed under controlled environmental conditions (21 °C, 12 h light – 12 h dark cycle) with free access to water and a standard diet *ad libitum*. It is important to note that the selection of these animals was based on the following considerations: (1) to eliminate the influence of cyclical physiological processes occurring in female rats, which could potentially affect the results of a long-term study (lasting up to 9 months), we utilized male rats as the experimental subjects; (2) the experimental studies on male Wistar rats commenced immediately after their sexual maturation, which occurs between 4 and 6 weeks of age; (3) previously published experimental data^13^ indicate that alcohol consumption in animals younger than 1 month does not lead to a statistically significant development of CAM. Each animal was housed individually and provided with a standard diet *ad libitum* in both quantity and composition. Body weight was measured daily for all animals across experimental groups throughout the study using Lab scales (“Dneproves FEH”, Ukraine). All procedures with laboratory animals complied with the ARRIVE guidelines. The use of animals was approved by the Biomedical Ethics Committee of the ESC “Institute of Biology and Medicine” of Taras Shevchenko National University of Kyiv (protocol no. 1 dated January 8, 2024) and performed following Article 26 of the Law of Ukraine “On the Protection of Animals from Cruelty” (no. 3447-IV, 21.02.2006), as well as European Union Directive of 22 September 2010 (2010/63/EU) for the protection of animals used for scientific purposes.

All tested rats were randomly divided into the following experimental groups:

- control (*n* = 36; 12 animals in each subgroup); animals received 100% drinking water;
- alcoholization (*n* = 36). Each rat was placed in a separate cage to receive 40% ethanol in drinking water, which meant that the animals did not have access to 100% water until they had consumed the dosed portion of ethanol^51^. The amount of ethanol consumed was calculated for 0.5% of the animal’s body weight. Recalculation of the ethanol dose was performed every 24 h throughout the experiment. The duration of AUD was 3 (*n* = 12), 6 (*n* = 12), and 9 (*n* = 12) months;
- alcoholization + C_60_ (*n* = 36; 12 animals in each subgroup). All tested rats, together with ethanol, were orally administered C_60_ fullerene at daily doses of 1 mg/kg. Recalculation of the C_60_ fullerene dose was performed every 24 h throughout the experiment. Control of the amount of C_60_ fullerene ingested was performed by denying the rats access to drinking 100% water until they fully consumed the applied drug^26,27,50^.

The dose of C_60_ fullerene was chosen as the most effective based on previous results^26,27,50^. Moreover, the total dose of 270 mg/kg in the experiment lasting 9 months is lower than the LD_50_ value, which was 600 mg/kg of body weight when administered orally to rats^52^ and 721 mg/kg when administered intraperitoneally to mice^53^. Also, it is important to note that after intravenous administration to mice, the radiolabeled C_60_ fullerenes predominantly accumulate in the blood, spleen, stomach, and liver and are excreted within 72 h, mainly with urine^47^. Finally, the histopathological and biochemical data show that C_60_ fullerene (at a daily dose of 1 mg/kg for 3, 6, and 9 months) reduces the level of liver damage in chronic alcohol intoxication of rats^50^. Therefore, a daily dose of 1 mg/kg is considered safe for biotesting.

Anesthesia of experimental rats was carried out by intraperitoneal injection of Zoletil (25 mg/kg, Virbac, France).

Experiments were conducted during the light phase, 4 to 5 h after alcohol administration. The interval between the induction of anesthesia and the start of the experiment did not exceed 15 min, and the total duration of each experiment was limited to 1 h.

Euthanasia of animals is conducted by administering an overdose of the anesthetic agent.

### Biomechanical and biochemical analyses

The contraction force of the *musculus gastrocnemius* was recorded with the Aurora Scientific (ASI) 305 C-LR Dual-Mode Lever System (USA) tensometric equipment.

In the distal part of the experimental *musculus gastrocnemius*, the tendon portion was cut and attached to the fixation sensors. In the L4-L5 segments, the ventral roots of the efferents were cut directly at their exit points from the spinal cord. The filaments of the severed ventral roots were fixed onto stimulating electrodes, and cyclic distribution of non-relaxation pulses was performed at a frequency of 50 Hz for 5 s. Control of the external load on the muscle was carried out using a system of mechanostimulators^26,27^.

In the framework of studying the biomechanics of muscle contraction, we analyzed the following indicators as markers of muscle dysfunction^25^:

1. integrated muscle power (S) is an area under the force curve, which is an indicator of the overall performance of the muscle under the applied stimulation pools. This value was calculated numerically using Origin 9.4 software and taken as 100% for the control group;
2. time of the beginning of the muscle force response (latent period; t_start_). This parameter indicates the level of pathological changes at the stages of the interaction of muscle myofilaments, dysfunction of the calcium pump, and the sarcoplasmic reticulum system, which are the result of myocyte membrane disruption;
3. time for the muscle force response to reach its initial level, taken as zero (t_0_). The increase in intramuscular collagen structures, the presence of nonfunctional muscle fibers, and the development of inflammation reduce the level of dynamic parameters of muscle contraction. Therefore, this parameter indicates a change in muscle stiffness associated with both an increase in connective tissue components (long-term pathology) and changes in intramuscular pressure (an inflammatory process);
4. time for the muscle contraction force to reach the maximum level (t_max_). Increasing this parameter triggers the CNS to recruit additional muscle fibers to ensure adequate muscle function. However, this can adversely impact the precise positioning of the joints^54^.

The concentration of GSH, CAT and SOD activities in the blood plasma of experimental rats, as the important markers of oxidative stress in the development of СAM^55,56^were determined using biochemical analyzers RNL-200 and JN-1101-TR2 (Netherlands).

Blood was collected from the experimental animals via femoral arterial catheterization^57^ and after that analyzed immediately using biochemical analyzers.

Our previous studies^50^ have shown significant pathological changes in the functioning of the organs of alcohol-exposed rats. Therefore, in the case of long-term СAM, the analysis of pro-antioxidant balance in muscle tissue may not be adequate, since during such a long period (up to 9 months), significant multi-organ disorders occur. Thus, we measured the values ​​of biochemical indicators in the blood plasma of experimental rats.

### MD simulation

We simulated 1–5, 10, 13, and 20 С_60_ fullerene clusters in ethanol solution. The initial structure of a single С_60_ fullerene was built by Avogadro software^58^. Nanoaggregates with 2–5, 10, 13, and 20 С_60_ fullerene units were constructed using package PackMol^59^. The same recourse was applied to put these clusters into the box with the solution of 1000 ethanol and 23,832 water molecules which corresponds to 11% of alcohol (a calculation model approximation). MD simulations are implemented in NAMD^60^ using force field CHARMM 36 with the type TYP3P of water atoms and Girifalco VDW parameters for C_60_ molecules^61^. Periodic boundary conditions are set in all directions. Firstly, С_60_ fullerene – ethanol/water systems were minimized during up to 10 ns in the NVT ensemble. Further systems were run for approximately 30 ns in the NPT ensemble. The size of the simulated box varied depending on the atom number in the systems to control the density of the ethanol solution, which was achieved at 0.98 g/cm^3^ corresponding to the reference value of 11% alcohol. Analysis of trajectories represented here is for the last 5 ns of running by VMD software (http://www.ks.uiuc.edu/Research/vmd/)^62^.

### Statistical analysis

The statistical evaluation of the experimental results was performed by the software package Statistica 8.0 (Dell, USA) using the procedure of analysis of variances (ANOVA) with mixed design^63^. Two between-group factors were supposed: (1) alcoholization (three levels – AUD lasting 3, 6, and 9 months); (2) C_60_ fullerene treatment (two levels – no and use of C_60_ fullerene). The Shapiro-Wilk W-test was used to test for normality. Levene’s test was used to assess the equality of variances across groups. Multiple pairwise comparisons between different groups and conditions were performed by Bonferroni *post-hoc* test. The differences between the groups were considered significant at *p* < 0.05. Each of the experimental force curves is the result of averaging 10 similar tests for each animal in the group. Each biochemical measurement was assayed in triplicate.

## Conclusion

The obtained results demonstrate a pronounced therapeutic effect of C_60_ fullerenes at the level of 16–50 ± 3% and 22–39 ± 2% for the studied biomechanical parameters of *musculus gastrocnemius* contraction and biochemical indicators of pro- and antioxidant balance in the blood of experimental rats, respectively, compared to the alcohol-exposed group against the background of CAM development. This indicates that the nanoantioxidants C_60_ fullerenes, effectively absorbing ROS, can improve the functional state of the muscular system during chronic alcohol intoxication. Finally, model calculations have shown that C_60_ fullerene nanoparticles present in ethanol, effectively binding its molecules, are also able to reduce the negative effect of alcohol on the functioning of the rat *musculus gastrocnemius*. Thus, C_60_ fullerenes can be considered promising drugs to reduce the extent of CAM symptoms developed, which requires further clinical trials.

## Data Availability

The datasets used and analyzed during the current study are available from the corresponding author upon reasonable request.
